# Targeted temperature management for adult out-of-hospital cardiac arrest: current concepts and clinical applications

**DOI:** 10.1186/s40560-016-0139-2

**Published:** 2016-04-27

**Authors:** Tatsuma Fukuda

**Affiliations:** Department of Emergency and Critical Care Medicine, Graduate School of Medicine, The University of Tokyo, 7-3-1, Hongo, Bunkyo-ku, Tokyo, 113-8655 Japan

**Keywords:** Out-of-hospital cardiac arrest, Cardiopulmonary resuscitation, Post-cardiac arrest syndrome, Targeted temperature management, Therapeutic hypothermia

## Abstract

Targeted temperature management (TTM) (primarily therapeutic hypothermia (TH)) after out-of-hospital cardiac arrest (OHCA) has been considered effective, especially for adult-witnessed OHCA with a shockable initial rhythm, based on pathophysiology and on several clinical studies (especially two randomized controlled trials (RCTs) published in 2002). However, a recently published large RCT comparing TTM at 33 °C (TH) and TTM at 36 °C (normothermia) showed no advantage of 33 °C over 36 °C. Thus, this RCT has complicated the decision to perform TH after cardiac arrest. The results of this RCT are sometimes interpreted fever control alone is sufficient to improve outcomes after cardiac arrest because fever control was not strictly performed in the control groups of the previous two RCTs that showed an advantage for TH. Although this may be possible, another interpretation that the optimal target temperature for TH is much lower than 33 °C may be also possible. Additionally, there are many points other than target temperature that are unknown, such as the optimal timing to initiate TTM, the period between OHCA and initiating TTM, the period between OHCA and achieving the target temperature, the duration of maintaining the target temperature, the TTM technique, the rewarming method, and the management protocol after rewarming. RCTs are currently underway to shed light on several of these underexplored issues. In the present review, we examine how best to perform TTM after cardiac arrest based on the available evidence.

## Introduction

The 2010 International Consensus on Cardiopulmonary Resuscitation and Emergency Cardiovascular Care Science with Treatment Recommendations (CoSTR) from the International Liaison Committee on Resuscitation (ILCOR) recommends therapeutic hypothermia (TH) (32–34 °C for 12–24 h) for comatose adult patients upon return of spontaneous circulation (ROSC) after out-of-hospital cardiac arrest (OHCA) with a shockable initial rhythm. It also states that TH may be considered for OHCA with a non-shockable initial rhythm or in-hospital cardiac arrest [1, 2]. This recommendation was based on two landmark randomized controlled trials (RCT) published in 2002 and supported by various subsequent studies [3–6]. However, a recently published large RCT raised questions about the effect of TH because that RCT showed no advantage for TH compared with normothermia (or fever control) [7].

In this review, we examine whether TH is beneficial after cardiac arrest and how best to perform targeted temperature management (TTM) after cardiac arrest on the basis of current evidence.

## Review

### Rationale for TTM

There are three distinct phases of brain injury in cardiac arrest [8, 9]. The first phase is intra-arrest ischemic injury due to no flow. In this phase, energy failure, ischemic depolarization of cell membranes, release of excitatory amino acids, and cytosolic calcium overload occur. Irreversible injury can be caused by them when ischemia is prolonged. The second phase is immediate reperfusion injury caused after ROSC. The resumption of oxidative phosphorylation can lead to reactive oxygen species production, mitochondrial calcium overload, and mitochondrial permeability transition, triggering cell death signaling. The third phase is delayed post-reperfusion injury. Secondary neuronal calcium overload, activation of pathologic protease, and altered gene expression and inflammation can occur and can last for several days. All three of these phases are potential targets for TTM.

Based on pathophysiological studies, hypothermia is believed to elicit neurological protection in multiple ways. Hypothermia decreases cerebral blood flow and cerebral oxygen consumption approximately by 7–8 % per 1 °C decline in temperature [10, 11]. Decreased cerebral metabolism protects the brain from further injury after anoxic injury [12, 13]. In addition, hypothermia affects two apoptotic cell death pathways. One is the intrinsic pathway under mitochondrial control, and the other is the extrinsic pathway signaled by an extracellular receptor [14]. Moreover, hypothermia reduces inflammation and free radical production [15]. Hypothermia can also prevent brain edema caused by blood-brain barrier disruption and increased vascular permeability following ischemia-reperfusion injury [16, 17].

Based on clinical studies, hypothermia was considered to improve outcomes after cardiac arrest. Two major RCTs that provided evidence of a benefit of TH were published in 2002 [3, 4].

In the European RCT, 275 adult comatose survivors after witnessed OHCA of presumed cardiac origin with shockable initial rhythm were enrolled [3]. Patients were randomly assigned to receive TTM (or TH) (target temperature, 32–34 °C; timing of initiation, at hospital after ROSC; treatment duration, 24 h from the start of cooling) or standard treatment with normothermia (Table 1). TTM group had more favorable neurological outcome (Glasgow-Pittsburgh cerebral performance category (CPC) of 1 or 2) within 6 months after OHCA than normothermia group (55 vs. 39 %; relative risk (RR), 1.40; 95 % confidence interval (CI), 1.08–1.81). In addition, 6-month mortality was lower in the TTM group than in the normothermia group (41 vs. 55 %; RR, 0.74; 95 % CI, 0.58–0.95). In this study, the body temperature of the normothermia group was not strictly managed. Therefore, the average body temperature in the normothermia group reached almost 38 °C because of the lack of fever control.

**Table 1:** Differences in detailed targeted temperature management protocol between guidelines and randomized controlled trials

Authors (published year)	Target temperature	Timing of initiation	Time to target temperature	Cooling techniques	Treatment duration	Sedatives	Neuromuscular blockades	Rewarming methods	Management after rewarming
HACA Study Group 2002 [3]	32–34 °C	After ROSC at hospital	Within 4 h after ROSC	External cooling device (TheraKool®) (if the goal was not achieved, ice packs were applied	24 h from cooling	Midazolam (0.125 mg/kg/h) and fentanyl (0.002 mg/kg/h) for 32 h	Pancuronium (0.1 mg/kg) every 2 h for a total of 32 h	Passive rewarming (over a period of 8 h)	/
Bernard, et al. 2002 [4]	33 °C	After ROSC in the ambulance	/	Ice packs (Coolcare®)	12 h after hospital arrival	Midazolam (2–5 mg) for 24 h	Vecronium (8–12 mg) for 24 h	Beginning at 18 h, actively rewarmed for the next 6 h by external warming with a heated-air blanket	Usual intensive care
CoSTR from ILCOR 2010 [1, 2] Guidelines from AHA, ERC 2010 [18, 19]	32–34 °C	Minutes to hours after ROSC	/	No single methods has proved to be optimal	12–24 h	/	/	/	Late hyperthermia (after rewarming post-hypothermia) should be identified and treated
Nielsen, et al. 2013 [7]	33 °C	After randomization at hospital, after ROSC	As rapidly as possible	Ice-cold fluids, ice packs, and intravascular or surface temperature-management devices at the discretions of the sites	28 h after randomization	Mandated until the end of the intervention period (36 h)	/	After 28 h, gradual rewarming to 37 °C (0.5 °C/h)	For unconscious patients below 37.5 °C until 72 h after cardiac arrest, with the use of fever control measures at the discretion of the sites

/ not described

In the Australian RCT, 77 adult comatose survivors after OHCA with shockable rhythm were enrolled [4]. Patients enrolled on odd-numbered days of the month were assigned to TTM (or TH) (target temperature, 33 °C; timing of initiation, in ambulance after ROSC; treatment duration, 12 h after hospital arrival), and patients enrolled on even-numbered days of the month were assigned to normothermia (Table 1). The TTM group had more favorable neurological outcomes at discharge from hospital than the normothermia group (49 vs. 26 %; *P* = 0.046). After adjustment for age and time from collapse to ROSC, the odds ratio (OR) for a favorable neurological outcome with TTM compared to normothermia was 5.25 (95 % CI, 1.47–18.76; *P* = 0.011). In this study, the body temperature of the normothermia group was relatively successfully maintained at 37.0 °C. However, the process of the randomization was inadequate because of the assignment in accordance with the day of the month, with patients assigned to hypothermia on odd-numbered days.

Although these two RCTs were not flawless, they are the basis for clinical practice guidelines that recommend TH after cardiac arrest issued by various organizations such as the American Heart Association (AHA), the European Resuscitation Council (ERC), and the Neurocritical Care Society (NCS) (Table 1) [18–20]. A Cochrane systematic review also supported these recommendations [5].

However, a large RCT published in 2013 has raised questions about the benefit of TH [7]. In this large RCT, 939 unconscious adult survivors after OHCA of presumed cardiac cause were enrolled irrespective of initial rhythm. Patients were randomly assigned to receive TTM of 33 °C (or TH) (target temperature, 33 °C; timing of initiation, at hospital after ROSC; treatment duration, 28 h from randomization) or TTM of 36 °C (or normothermia with strict fever control) (Table 1). At the end of the trial, the mortality rates in both groups were similar (50 vs. 48 %; hazard ratio (HR) with a temperature of 33 °C, 1.06; 95 % CI 0.89–1.28; *P* = 0.51). At the 180-day follow-up, neurological outcomes were also similar in both groups, according to a CPC of 3–5 (54 vs. 52 %; RR, 1.02; 95 % CI 0.88–1.16; *P* = 0.78) or a modified Rankin scale of 4–6 (52 vs. 52 %; RR, 1.01; 95 % CI 0.89–1.14; *P* = 0.87). The authors concluded that TTM of 33 °C did not confer any benefit for unconscious survivors after OHCA relative to TTM of 36 °C.

TTM study makes it difficult to judge whether outcomes after OHCA are improved by hypothermia. However, TTM study and previous two RCTs differed not only in their implementation of strict fever control in the control group but also in some other details (Table 1). Therefore, the details of practical methods for TTM should also be examined.

### Practical methods for TTM

**Fig. 1:**
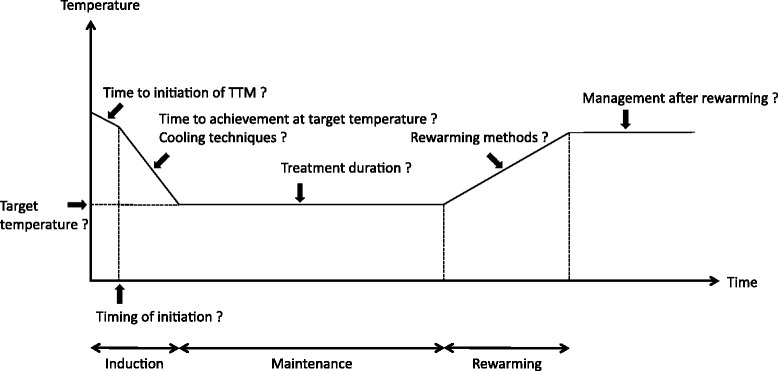
Time course of targeted temperature management

There are three phases in TTM (mainly TH): induction, maintenance, and rewarming. Unsolved issues remain in each phase, including the appropriate target temperature, the timing of initiation, the duration from cardiac arrest to initiation of TTM or the achievement of target temperature, the treatment duration, the TTM technique, and the rewarming method (Fig. 1).

#### Target temperature

The optimal target temperature for neurologically favorable outcomes after OHCA is unclear at this time. Although mild hypothermia (32–34 °C) has been strongly recommended by various organizations on the basis of the two RCTs published in 2002, several subsequent studies, and pathophysiological findings, the recently published RCT comparing TTM of 33 °C with TTM of 36 °C showed no beneficial effect of mild hypothermia compared with normothermia (with strict fever control) [1–7, 10–19]. This could mean that strict fever control alone may be sufficient to improve neurological outcomes among OHCA compared with mild hypothermia, or that the optimal target temperature may be lower than 33 °C. However, the differences in the main results of the three RCTs that compared TH with normothermia may be caused by differences in their implementations of TTM other than the target temperature (Table 1). To determine the optimal target temperature for improving neurological outcomes after OHCA, further study will be required; comparing a range of target temperature levels without varying other aspects of TTM. An RCT comparing TTM at 31 °C and TTM at 34 °C (CAPITAL CHILL (NCT02011568)) is currently recruiting, and it may provide further clarification.

#### Timing of initiation and duration from cardiac arrest to initiation or target temperature

The optimal timing for initiating TTM is unknown. In addition, the effect on outcomes of delay between the onset of cardiac arrest and initiating TTM or achieving the target temperature is not well understood.

Animal experiments have shown that earlier initiation of TH increases the efficacy of TH [21, 22] and that survival rates are high and brain injury is mild when TH is initiated during the intra-arrest period before ROSC [23, 24]. However, similar results have not always been obtained in studies involving human subjects. A registry study in Scandinavia including 986 TH-treated OHCA patients, the largest-scale study of this issue, did not reveal any relationship between neurologic outcomes and the length of time from cardiac arrest until the initiation of TH (median 90 min, *p* = 0.48) or until the target temperature was achieved (median 260 min, *p* = 0.91) [6]. However, according to a study in the USA using data from the International Cardiac Arrest Registry (INTCAR), which included 172 OHCA patients treated with TH, every 5-min delay in initiating TH (mean 94.4 min) was associated with a worsening in neurologic outcomes at intensive care unit (ICU) discharge (OR 1.06, 95 % CI 1.02–1.10), at hospital discharge (OR 1.06, 95 % CI 1.02–1.11), and 1 month after hospital discharge (OR 1.08, 95 % CI 1.03–1.13) [25]. Furthermore, neurologic outcomes 1 month after discharge worsened with every 30-min delay in achieving the target temperature (OR 1.17, 95 % CI 1.01–1.36). In another study in the USA, which included 140 OHCA patients who had achieved ROSC, a 20 % increase in the risk of death (95 % CI 4–39 %) was observed for every hour of delay in initiation of TH [26]. In a study in Germany that included 49 successfully resuscitated OHCA and in-hospital cardiac arrest (IHCA) patients, neurologic outcomes worsened for every hour delay in reaching the target temperature (OR 0.69, 95 % CI 0.51–0.98) [27]. It is necessary to note that comparatively long delays, approximately 80–150 min from cardiac arrest to initiation of TH and approximately 260–410 min from cardiac arrest to achievement of target temperature, were observed in these studies.

Several studies have examined the feasibility, safety, and efficacy of pre-hospital TH with the purpose of more quickly initiating TH and achieving the target temperature. The safe and effective introduction of TH appears to be feasible not only after ROSC [28–31] but also before ROSC (intra-arrest or during resuscitation) [32, 33].

There have been many RCTs investigating the relationship between pre-hospital TH after ROSC and outcomes after OHCA [34–36], and meta-analyses have also been conducted [37, 38]. In all studies, pre-hospital TH decreased core temperature on hospital arrival and reduced the time to target temperature, but improvements in survival rates and neurologic outcomes were not shown.

Animal experiments and clinical studies have shown decreases in myocardial infarction size and increases in ROSC rates when TH was initiated before ROSC (intra-arrest) [39, 40], and a systematic review showed that, although clinical data are limited, intra-arrest TH improved not only ROSC rates but also survival rates and favorable neurologic outcomes [41]. Two large RCTs examining pre-hospital intra-arrest TH by intranasal cooling (PRINCESS (NCT01400373)) or by rapid infusion of cold normal saline (RINSE (NCT01173393)) are ongoing.

Although there is currently little evidence supporting earlier initiation of pre-hospital TH after ROSC, its introduction before ROSC may be effective; thus, the results of the RCTs currently in progress will provide important clarification.

#### Treatment duration

The 2010 AHA guidelines states that the optimal duration of TH is at least 12 h and may be more than 24 h [18]. This recommendation is based on two RCTs that showed beneficial effects of TH for adult OHCA with a shockable initial rhythm [3, 4]. Although TH for up to 72 h has been used safely in newborns, the effect of longer TH duration on outcomes for adult OHCA has not been studied [42, 43]. An RCT of prolonged TH (at 32–34 °C for 24 vs. 48 h) (TTH48 (NCT01689077)) is now recruiting, and it may provide further information.

#### TTM techniques

Common methods used for cooling include rapid infusion of ice-cold IV fluid, ice packs, water-circulating blankets, air-circulating blankets, water-circulating gel-coated pads, and intravascular cooling devices (Table 2). Moreover, there are methods that use cardiopulmonary bypass [44], nasopharyngeal cooling [45], transnasal evaporative cooling [33, 46], cold-air tents [3], and cooling helmets [47, 48], among others. The CoSTR from ILCOR lists rapid infusion of ice-cold IV fluid and ice packs as feasible, safe, and simple introduction methods that do not require any specialized devices [1, 2].

**Table 2:** Cooling techniques

	Rapid infusion of ice-cold IV fluid and ice packs	Water-circulating blankets	Air-circulating blankets	Water-circulating gel-coated pads	Intravascular cooling devices
Induction phase					
Simpleness					
Pre-hospital	◯	×	×	×	×
After hospital arrival	◯	△	△	△	×
		Specialized devices	Specialized devices	Specialized devices	Specialized devices intravascular catheterization
Non-invasiveness	◯	◯	◯	◯	×
					intravascular catheterization
Cooling rate	×	◯	×	◯	◯
	0.32 ± 0.24 °C/h	1.33 ± 0.63 °C/h	0.18 ± 0.20 °C/h	1.04 ± 0.14 °C/h	1.46 ± 0.42 °C/h
Maintenance phase					
Stability^a^	×	×	×	×	◯
	69.8 ± 37.6 %	50.5 ± 35.9 %	74.1 ± 40.5 %	44.2 ± 33.7 %	3.2 ± 4.8 %
Convenience	×	△	△	◯	◯
	Frequent manual exchange	Manual control	Manual control	Automated control	Automated control
Inexpensiveness	◯	△	△	×	×
		Specialized devices	Specialized devices	Specialized devices	Specialized devices

^a^The percentage of time the patient’s temperature was out of range more than 0.2 °C below or above the target temperature

Reference: [49] Hoedemaekers CW, Ezzahti M, Gerritsen A, van der Hoeven JG. Comparison of cooling methods to induce and maintain normo- and hypothermia in intensive care unit patients: a prospective intervention study. Crit Care 2007; 11: R91

In an RCT, the following five cooling methods were compared in 50 ICU patients who required strict TTM, including 16 OHCA patients and 4 IHCA patients: (1) rapid infusion of 30 ml/kg cold fluids and ice-cold packs (conventional cooling), (2) water-circulating blankets, (3) air-circulating blankets, (4) water-circulating gel-coated pads, and (5) intravascular cooling devices [49] (Table 2). Temperature decline was greater with the water-circulating blankets (1.33 °C/h), water-circulating gel-coated pads (1.04 °C/h), and intravascular cooling devices (1.46 °C/h) compared to conventional cooling (0.32 °C/h) and the air-circulating blankets (0.18 °C/h) (*p* < 0.05). Moreover, the percentage of time for which the patient’s temperature was more than 0.2 °C below or above the target temperature was significantly lower with the intravascular cooling device (3.2 %) than with other methods (water-circulating gel-coated pad, 44.2 %; water-circulating blanket, 50.5 %; conventional cooling, 69.8 %; air-circulating blanket, 74.1 %; *p* < 0.05). Based on these findings, an intravascular cooling device may be an efficient way to achieve the target temperature earlier and to maintain a stable temperature.

Two RCTs have been conducted to evaluate the clinical impact of intravascular cooling on outcomes after OHCA. One RCT compared invasive advanced internal cooling (CoolGard®) with non-invasive advanced external cooling (ArcticSun®) [50], and the other RCT compared invasive advanced internal cooling (CoolGard®) with non-invasive basic external cooling (using fans, cooling tents if possible, and ice packs) [51]. These two RCTs showed no beneficial effects of intravascular cooling on survival or favorable neurological outcomes after OHCA. However, the target temperature was more strictly maintained with intravascular cooling. The time to achieve the target temperature with advanced internal cooling (CoolGard®) was similar to that with advanced external cooling (ArcticSun®) but significantly shorter than that with basic external cooling. Bleeding complications were more frequent with intravascular cooling.

Thus, it appears that intravascular cooling devices are beneficial for reducing time to target temperature and improving strict maintenance of the target temperature, but this does not always lead to improved outcomes. Currently, there is insufficient evidence to recommend any specific cooling method. Therefore, it is necessary to fully understand the advantages and disadvantages of each cooling method and consider which combination of methods is appropriate for each facility.

When TH is initiated, cooling methods that do not require specialized devices, such as rapid infusion of ice-cold IV fluid and ice packs, as described in the ILCOR guidelines [1, 2], may be useful because they can be implemented anytime and anywhere (including pre-hospital, during transport, or during resuscitation) and are safe and inexpensive. Conversely, during the maintenance phase, the selection of a cooling method that matches the available budget, manpower, and equipment of each facility may be desirable.

#### Rewarming

According to the ERC guidelines, because plasma electrolyte concentrations and effective intravascular volumes and metabolic rates are likely to change suddenly, it is recommended to perform rewarming slowly [19]. The recommended rate for rewarming in the ERC Hypothermia After Cardiac Arrest Registry (ERC HACA-R) is 0.25–0.5 °C/h [52].

In a retrospective cohort study including 128 patients treated with TH after cardiac arrest, the authors examined rewarming method (active vs. passive), rewarming speed (≧0.5 °C/h vs. <0.5 °C/h), and the relationship between fever (>38 °C) and poor outcomes. The odds ratios for poor outcomes after adjustment for confounders were as follows: active rewarming, 1.51 (95 % CI 0.64–3.58, *p* = 0.35); rewarming speed ≧0.5 °C/h, 2.61 (95 % CI 0.88–7.73, *p* = 0.08); fever, 0.64 (95 % CI 0.31–1.30, *p* = 0.22) [53]. There were no significant associations between rewarming method and outcomes in this study. It is expected that RCTs will also be performed to investigate optimal rewarming methods.

The AHA guidelines recommend treatment of hyperthermia following rewarming of the patient through TH [18]. This is based on several studies that have shown a relationship between hyperthermia in post-cardiac arrest syndrome (PCAS) and poor outcomes [54–57]. Based on these studies, hyperthermia should be avoided for 48–72 h after ROSC.

In the Penn Alliance for Therapeutic Hypothermia (PATH) Registry, a multicenter US clinical registry that includes 167 patients who survived 24 h after post-TTM rewarming, the relationships between rebound pyrexia (defined as temperature >38 °C) and clinical outcomes were examined [58]. After post-TTM rewarming, pyrexia was observed in 41 % of patients, and the median temperature was 38.7 °C. There were no significant differences between the pyrexia group and the no pyrexia group in survival rate (54 vs. 52 %, *p* = 0.88) or favorable neurologic outcome (70 vs. 82 %, *p* = 0.21). However, when the authors compared marked pyrexia (greater than the median pyrexia of 38.7 °C) with no or milder pyrexia (below the median), the survival rate did not differ (40 vs. 56 %, *p* = 0.16), but marked pyrexia was associated with more frequent poor neurological outcomes (58 vs. 80 %, *p* = 0.04). Based on these findings, it may be that there is a relationship between higher pyrexia after post-TTM rewarming and more severe brain injury or that there is a threshold temperature above which an effect on outcomes is detectable. However, because their study design can only indicate an association, not a causal relationship, it is difficult to resolve these issues decisively. Further study is necessary to identify the maximum safe temperature after post-TTM rewarming, and the duration for which temperature management should be continued, and the importance of avoiding hyperthermia after completion of rewarming.

### Which patients benefit the most from TTM

In this review, we mainly examined how best to perform TTM after OHCA. However, we also know very little about which patients benefit the most from TTM. What we do know is that TTM seems to have the most impact on favorable outcomes among patients with an initial shockable rhythm [3, 4, 59]. Several studies have examined whether the effectiveness of TTM might depend on the time, such as no-flow time, low-flow time, or total down-time [59–62], or on the severity of anoxic-ischemic injury [63–65]. Well-designed studies will be required to identify who can benefit the most from TTM.

## Conclusions

It is not yet possible to identify the most appropriate practical method for TTM after OHCA. A recently published large RCT showed no advantage for TTM of 33 °C in comparison with TTM of 36 °C, but it would be premature to discard the numerous previous studies and the pathophysiology that support the efficacy of TH. On the other hand, this large RCT also confirmed that strict TTM is essential. Although it remains undetermined whether TH should be performed, it is necessary to surely manage temperature to avoid hyperthermia. RCTs concerning the optimal target temperature, optimal timing of initiation, and optimal duration of treatment are currently in progress. To determine whether TH is beneficial after cardiac arrest and how best to perform TTM after cardiac arrest, the ongoing RCTs deserve careful attention.

## Abbreviations

AHA: American Heart Association
CI: confidence interval
CoSTR: Internatinal Consensus on Cardiopulmonary Resuscitation and Emergency Cardiovascular Care Science with Treatment Recommendations
CPC: Glasgow-Pittsburgh cerebral performance category
ERC: European Resuscitation Council
HR: hazard ratio
ICU: intensive care unit
IHCA: in-hospital cardiac arrest
ILCOR: International Liaison Committee on Resuscitation
NCS: Neurocritical Care Society
NSE: neuron-specific enolase
OHCA: out-of-hospital cardiac arrest
OR: odds ratio
PCAS: post-cardiac arrest syndrome
RCT: randomized controlled trial
ROSC: return of spontaneous circulation
RR: relative risk
TH: therapeutic hypothermia
TTM: targeted temperature management
